# Independent risk factors for diversion colitis: a retrospective case-control study

**DOI:** 10.1590/1806-9282.20241590

**Published:** 2025-05-02

**Authors:** Di Wu, Bin Wang, Hao Yang

**Affiliations:** 1 Air Force Medical University, Tangdu Hospital, Department of General Surgery – Xi'an, China.; 2 The 964th Hospital of the Joint Logistics Support Force, Department of Endocrinology – Changchun, China.; 3 The 964th Hospital of the Joint Logistics Support Force, Department of Radiology – Changchun, China.

**Keywords:** Colitis, Colorectal surgery, Rectal cancer, Risk factor

## Abstract

**OBJECTIVE:**

The aim of this study was to investigate independent risk factors for diversion colitis induced by the surgical interruption of fecal flow in the non-functional colon.

**METHODS:**

We performed a retrospective study with 163 patients who underwent low anterior resections and created prophylactic ileostomies for rectal cancer between January 2014 and June 2023 at the Department of General Surgery, Air Force Medical University Tangdu Hospital. Colonoscopy results of the non-functional region of the distal colon and clinical variables were collected, including age, sex, body mass index, pathological tumor node metastasis staging, ileostomy method, diversion time, receiving radiotherapy or chemotherapy or not, suffering from preoperative inflammatory bowel disease or postoperative anastomotic leakage or not. Diagnosis of diversion colitis based on the results of the patients’ colonoscopy results. Univariate analysis and multivariate analysis of diversion colitis-related risk factors were performed subsequently.

**RESULTS:**

The morbidity of diversion colitis is 53.4% (87/163) in our study. Multivariate analysis showed that risk factors for diversion colitis included single-lumen prophylactic ileostomy (63.2 vs. 30.3%, OR 4.481, 95%CI 1.897–10.584, p<0.001), diversion time ≥90 days (79.3 vs. 40.8%, OR 4.474, 95%CI 1.849–10.826, p<0.001), inflammatory bowel disease (17.2 vs. 3.9%, OR 7.491, 95%CI 1.839–30.507, p=0.005), radiotherapy (58.6 vs. 42.1%, OR 0.515, 95%CI 0.196–1.352, p=0.178).

**CONCLUSION:**

These findings suggest that single-lumen prophylactic ileostomy, diversion time, and inflammatory bowel disease are independent risk factors for diversion colitis.

## INTRODUCTION

With the gradual promotion of total mesorectal excision, the widespread performing of laparoscopic surgery, and stapler technology in clinical practice, the sphincter-preserving rate of patients with low rectal cancer has significantly improved^1,2^. However, the accompanying increased risk of anastomotic leakage has become an important issue that endangers safety and decreases patients’ quality of life^3-5^. Prophylactic ileostomy (PI) has become an important choice for surgeons to reduce the risk of anastomotic leakage and reoperation rate after low anterior resection^6^. However, while PI plays a protective role in anastomotic leakage, its complications also need sufficient attention, such as diverting colitis (DC)^7,8^. DC is a non-specific inflammation of the non-functional region of the distal colon induced by surgical interruption of fecal flow through a stoma^7^. The pathogenesis of DC is still unclear. Endoscopic findings include edema, mucosal hemorrhage, and so on, and clinical symptoms include mucous discharge, tenesmus, bleeding, abdominal pain, and so on^7^. The report points out that the trigger for DC is the interruption of fecal flow^7^.

## METHODS

A total of 766 consecutive patients had surgeries for rectal cancer at the Department of General Surgery, Air Force Medical University Tangdu Hospital, between January 2014 and June 2023. This study has been approved by the Ethical Committee of Air Force Medical University Tangdu Hospital (approval number: 21-KY-14-XW-23) and was conducted in accordance with the Declaration of Helsinki after obtaining informed consent from the patients or their family members. Rectal cancer was defined as a tumor 15 cm or less from the anal verge measured with a rigid colonoscopy. The surgical indications included clinical T1–3 lesions based on pelvic computed tomography and magnetic resonance imaging. The eligible patients were performed PI after LAR, with a pathological tumor node metastasis staging (pTNM) class 1–3. The initial exclusion criteria included no ileostomy creation, no colonoscopy results, loss of follow-up, death, and local recurrence. For patients, the stoma closure can be performed 1–6 months after the PI. Before the stoma closure, they underwent endoscopy. A single endoscopist reviewed the colonoscopy results and diagnosed whether patients had suffered from DC or not based on the endoscopic findings such as edema and mucosal hemorrhage. Other clinical variables were collected, including age, sex, body mass index (BMI), pTNM, ileostomy method, diversion time, receiving radiotherapy or chemotherapy or not, suffering from preoperative inflammatory bowel disease (IBD) or postoperative anastomotic leakage or not. Diversion time means the time interval between PI and colonoscopy before stoma closure. PI is divided into the "single-lumen PI" group and the "double-lumen PI" group. Single-lumen PI: Cut off the ileum at a distance of 20 cm from the ileocecal region, and pull the proximal end out of the abdominal wall for ileostomy; Double-lumen PI: Take the ileum out of the abdominal wall at a distance of 20 cm from the ileocecal region for ileostomy without cutting off the ileum. Radiotherapy and chemotherapy for rectal cancer refer to the latest guidelines^9^.

### Statistical analysis

Kolmogorov-Smirnov tests were performed on all continuous variables. If normally distributed, described by mean±standard deviation, an independent-samples t-test was used for between-group analysis; for those not normally distributed, median and interquartile range were used, and the Mann-Whitney U test was used for between-group comparisons. The categorical variables are represented by numbers (%), and comparisons between groups were made using the Pearson χ^2^ test or Fisher's exact test. The association between risk factors and DC was assessed using the chi-square test (χ^2^) and unadjusted odds ratio (OR), along with the p-value that was reported for univariate analysis. Risk factors for DC were assessed using binary logistic regression analysis (entry and exit criteria of p=0.05 and p=0.20, respectively), with the adjustment of the variables including single-lumen PI, diversion time v90 days, IBD, and radiotherapy. Subsequently, multivariable logistic regression was utilized to obtain the adjusted OR and confidence intervals (CIs) for variable estimates. A two-sided p-value <0.05 was used to declare statistical significance.

## RESULTS

The final retrospective study included 163 patients. The patients’ baseline characteristics are listed in Table 1. Among the enrolled patients, 87 were in the DC group and 76 were in the non-DC group. There was no statistical significance between the two groups in terms of age (56.8±12.9 vs. 57.0±12.8, p=0.903), BMI (23.9±3.1 vs. 23.8±3.5, p=0.845), female (35.6 vs. 35.5%, p=0.989), chemotherapy (77.0 vs. 76.3%, p=0.917), anastomotic leakage (4.6 vs. 3.9%, p=1.000), and pTNM staging (19.5 vs. 18.4%, 39.1 vs. 39.5%, 41.4 vs. 42.1%, p=0.983).

**Table 1:** Baseline characteristics of patients by diversion colitis.

Baseline characteristics		Total (163)	DC (87)	Non-DC (76)	p-value
Age (years)		56.9±12.8	56.8±12.9	57.0±12.8	0.903
Female, n (%)		58 (35.6%)	31 (35.6%)	27 (35.5%)	0.989
BMI (kg/m^2^)		23.8±3.3	23.9±3.1	23.8±3.5	0.845
Single-lumen PI, n (%)		78 (47.9%)	55 (63.2%)	23 (30.3%)	<0.001
Diversion time ≥90 days, n (%)		100 (61.3%)	69 (79.3%)	31 (40.8%)	<0.001
IBD, n (%)		18 (11.0%)	15 (17.2%)	3 (3.9%)	0.007
Radiotherapy, n (%)		83 (50.9%)	51 (58.6%)	32 (42.1%)	0.035
Chemotherapy, n (%)		125 (76.7%)	67 (77.0%)	58 (76.3%)	0.917
Anastomotic leakage, n (%)		7 (4.3%)	4 (4.6%)	3 (3.9%)	1.000
pTNM staging					
	I	31 (19.0%)	17 (19.5%)	14 (18.4%)	0.983
	II	64 (39.3%)	34 (39.1%)	30 (39.5%)	
	III	68 (41.7%)	36 (41.4%)	32 (42.1%)	

DC: diversion colitis; BMI: body mass index; IBD: inflammatory bowel disease; pTNM: pathological tumor node metastasis; PI: prophylactic ileostomy.

Univariate analysis showed that DC has a higher morbidity in patients with single-lumen PI (63.2 vs. 30.3%, OR 3.961, 95%CI 2.057–7.627, p<0.001), diversion time ≥90days (79.3 vs. 40.8%, OR= 5.565, 95%CI 2.786–-11.112, p<0.001), IBD (17.2 vs. 3.9%, OR 5.069, 95%CI 1.407—18.263, p=0.013), and radiotherapy (58.6 vs. 42.1%, OR 1.948, 95%CI 1.044–3.636, p=0.036) (Table 2).

**Table 2:** Univariate analysis of diversion colitis-related risk factors.

Source	OR	95%CI
Single-lumen PI, n (%)	3.961	(2.057–7.627)
Diversion time ≥iv days, n (%)	5.565	(2.786–11.112)
IBD, n (%)	5.069	(1.407–18.263)
Radiotherapy, n (%)	1.948	(1.044–3.636)

PI: prophylactic ileostomy; IBD: inflammatory bowel disease; OR: odds ratio; CI: confidence interval.

**Figure 1:**
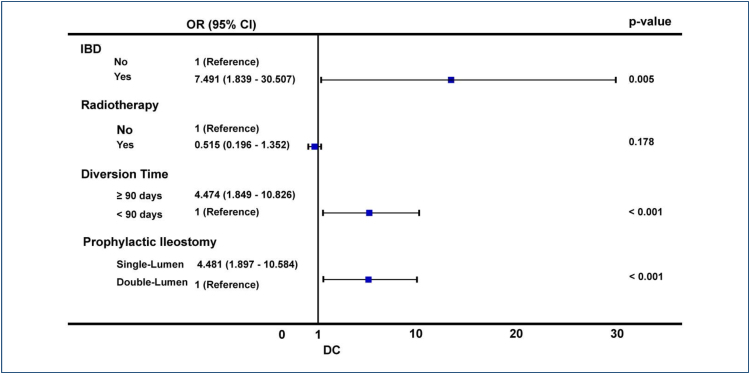
Multivariate analysis of diversion colitis-related risk factors.

The variables with p<0.20 in univariate analysis were selected for multivariable analysis. Multivariate analysis showed that risk factors for DC included single-lumen PI (63.2 vs. 30.3%, OR 4.481, 95%CI 1.897–10.584, p<0.001), diversion time ≥90days, (79.3 vs. 40.8%, OR= 4.474, 95%CI 1.849–-10.826, p<0.001), IBD (17.2 vs. 3.9%, OR 7.491, 95%CI 1.839–30.507, p=0.005), and radiotherapy (58.6 vs. 42.1%, OR 0.515, 95%CI 0.196–1.352, p=0.178) (Figure 1). Finally, single-lumen PI, diversion time, and IBD are independent risk factors for DC.

## DISCUSSION

Stoma-induced DC can cause patients to experience a series of symptoms such as abdominal pain, mucous discharge, bleeding, tenesmus, and diarrhea after the stoma closure. A colonoscopy examination reveals edema, mucosal hemorrhage, and so on in the non-functioning region of the distal colon^7^. The occurrence and severity of DC reported in the literature are time-dependent, gradually worsening with the increase of diversion time. Literature has reported a morbidity of approximately 70–100% for DC^7,10^. In our study, the morbidity was 53.4%, which may be related to the early stoma closure and the conduction of double-lumen PI. Previous studies have shown that DC can reduce patients’ quality of life^11^.

The pathogenesis of DC may be as follows: prolonged fecal flow diversion leads to a decrease in anaerobic bacterial concentration and an increase in nitrate-reducing bacteria in the non-functional colon, resulting in a toxic level of nitric oxide produced by its metabolism. The toxic level of nitric oxide leads to DC^12,13^. These situations can be improved through microbiota transplantation.^14,15^ Some scholars also believe that ischemia is the cause of DC^16^, based on the reduction of short-chain fatty acids (SCFAs), which are produced by normal intestinal bacteria. SCFAs have the effect of relaxing vascular smooth muscle, and insufficient SCFAs may cause vasoconstriction of the pelvic artery, leading to insufficient blood supply to the colon. After local treatment with SCFAs for some time, some DC patients can improve their clinical symptoms^17^. At present, there is no consensus on the exact pathogenesis of DC; however, there is a basic consensus that prolonged fecal flow diversion is a risk factor for DC. Traditionally, it is believed that the stoma closure should be performed 8–12 weeks after an ostomy; however, it is usual that this period extends beyond 12 months^18^. If the examination results show complete healing of the anastomosis, no local recurrence, no anastomotic stenosis, no anastomotic leakage, and no other complications, then the stoma closure should be performed immediately^18,19^. Some scholars also believe that early stoma closure can reduce the morbidity of stoma-related complications and patients’ discomfort^20^. O’Sullivan et al. reported that stoma closure was feasible <14 days after the ostomy, but the morbidity of reoperations and postoperative ileus was higher^21^. Other studies have also found that there is no significant difference between early and late stoma closure in the mortality of complications^18^. The report by Nelson et al. suggested that early stoma closure did not only increase the risk of postoperative complications but also reduced the cost of ostomy care and improved patients’ quality of life^22^. In short, the results of reports on whether to choose early or late stoma closure are contradictory.

The purpose of ostomy is to divert the fecal flow out of the body through the artificially established stoma, to reduce the tension of the anastomotic region in the Phase I operation, and to reduce the morbidity of anastomotic leakage and the reoperation rate^1,6^. Szczepkowski et al. reported that the morbidity of DC was not related to the ostomy method^23^. However, they only compared single-lumen PI to single-lumen colostomy, and the conclusion that DC is not related to the ostomy method is not accurate. Single-lumen PI completely interrupts intestinal continuity, thereby achieving the goal of fecal flow diversion. A double-lumen PI may not completely interrupt fecal flow, resulting in a small amount of fecal flow nourishing a portion of the distal intestine. Maybe for this reason, our study indicates that the morbidity of DC after double-lumen PI is lower than that of single-lumen PI.

IBD mainly includes ulcerative colitis and Crohn's disease. The morbidity of DC in patients without a preoperative diagnosis of IBD is 70–74%, while the morbidity in patients with a preoperative diagnosis of IBD is 91%^7^. Korelitz et al. reported that the morbidity of DC in patients who were also diagnosed with IBD before the operation was even as high as 100%^10^ after fecal flow was interrupted. Our study shows that IBD is a risk factor for DC, which may be due to the rapid change in gut microbiota caused by fecal flow diversion, accelerating the destruction of the already fragile gut microbiota ecology in patients with IBD and leading to the occurrence of DC.

Radiotherapy is a very important part of the treatment for colorectal cancer^24,25^. In our study, radiotherapy had significant differences in the univariate analysis of risk factors, but it was not an independent risk factor for DC. The reason may be that 49.4% of patients with radiotherapy had a diversion time ≥ I days, and 47.0% had a single-lumen PI. We have also retrieved relevant literature, and there have been no reports that radiotherapy is an independent risk factor for DC. Due to the above results, we need to follow up on this study, increase the sample size, and continue to explore whether radiotherapy is an independent risk factor for DC. In summary, single-lumen PI, diversion time, and IBD are independent risk factors for DC. Our study indicated that for patients with high-risk factors such as IBD implementing, double-lumen PI may be beneficial, and whether single-lumen or double-lumen PI that patients undergo, stoma closure implemented within 3 months may be appropriate.

Some limitations need to be considered: (1) this study is a single-center study with a relatively small sample size, which can lead to selective bias; (2) our study is retrospective, and we cannot collect data on changes in gut microbiota and metabolic products between the two groups of patients, which limits the revelation of the pathogenesis of DC; and (3) multicenter and prospective studies are needed to confirm our findings further.
